# A rapid method for separating and concentration of food-borne pathogens using elution from ready-to-eat vegetables

**Published:** 2018-12

**Authors:** Safieh Rajabzadeh, Masoumeh Bahreini, Mohammad Reza Sharifmoghadam

**Affiliations:** 1 Department of Food Sciences and Technology, Resarch Institute of Food Sciences and Technology, Mashhad, Iran; 2 Department of Biology, Faculty of Sciences, Ferdowsi University of Mashhad, Mashhad, Iran

**Keywords:** Rapid detection, Elution, Multiplex polymerase chain reaction, Food-borne pathogens, Ready-to-eat vegetables

## Abstract

**Background and Objectives:**

Traditional culture methods for detection of food-borne pathogens, a major public health problem, are simple, easily adaptable and very practical, but they can be laborious and time consuming. In this study, we eliminated culturing steps by developing a new separation method and therefore, decreased the detection time of food-borne pathogens (*Salmonella enterica* serovar Typhimurium, *Escherichia coli* O157:H7 and *Listeria monocytogenes*) to a few hours.

**Materials and Methods:**

We used alkaline water and different alkaline buffers to elute bacteria from the lettuce surface as a model for ready-to-eat vegetables. Buffers used were as follows: 1) 0.05 M glycine; 2) 0.05 M glycine −100 mM Tris base −1% (w/v) beef extract; 3) buffer peptone water; 4) buffer phosphate saline. Buffers were adjusted to pH of 9, 9.5 and 10. In order to elute the bacteria, the lettuce pieces were suspended into buffers and shacked for 30, 45 and 60 min. Moreover, a multiplex PCR method for the simultaneous detection of food-borne pathogens was performed.

**Results:**

The results showed that buffer peptone water at pH 9.5 for 45 min have high ability to elute bacteria from the lettuce surface and the bacteria can be detected using multiplex PCR.

**Conclusion:**

We developed a new rapid and efficient method for simultaneous separation of food-borne pathogens. This method eliminates culturing stages and permits the detection and identification of target pathogens in a few hours.

## INTRODUCTION

In the recent years, the growing consumption of fresh and ready-to-eat vegetables has caused an increase in the number of food-borne disease outbreaks and wastewater irrigation is an issue of concern to public health and the main source of contamination of vegetables (1). Raw and minimally processed ready to eat vegetables could be hazardous for the safety and health of the consumers. According to Korean FDA, the number of food-borne diseases in 2008 increased 3.8-fold compared to 2003 (2). In Europe, for instance, human food-borne cases and outbreaks were reported yearly (3–5). The global incidence of foodborne outbreaks is difficult to estimate, although authors generally agree with the estimate that the percentage of the population affecting from food-borne disease each year could be up to 30% in industrialized countries and it could be worse in developing countries (6). Therefore, there is an urgent and serious need for a rapid, sensitive and reliable method to detect food-borne pathogens, especially for high-risk organisms such as *Salmonella, Listeria monocytogenes* and *Escherichia coli* O157:H7 (7). It is also necessary to develop a new method, which could considerably decreases the spending time and the cost.

Detection of foodborne pathogens based on conventional culture methods, culture media and biochemical kits, are simple, easily adaptable, very practical, and generally inexpensive, however, they can be laborious and time-consuming (7–9).

Advances in technology have led to the use of molecular methods and new nanomaterials to detect pathogens. Molecular techniques would significantly decrease the resources required in routine laboratory operations, and would enhance the overall efficiency of detection in food supervision and inspection (2, 10–17). In these methods, however, due to the importance of presence of even 1 cfu/25 g of some pathogens in foodstuffs, especially *Salmonella* spp., *Listeria monocytogenes* and *E. coli* O157:H7, the pre-enrichment and enrichment stages are used (10). These methods are laborious and increase the detection time to 2–3 days (7, 2, 18). The incubation time of 6 hours is the minimum incubation period that has been used by Thapa and co-workers (2).

Recently, food-borne viruses have become an important food safety concern and various studies have dealt with the development of standardized methods for detection of enteric viruses in foods. Viruses are able to attach to food surfaces through their negatively charged surface proteins. Changing the pH from neutral to alkaline alters the electrical charge that separates the viral particles from the food surface (19, 20). Many papers have been published using alkaline pH for elution of food-borne viruses from the vegetable and food surfaces (21–23).

Bacteria, like viruses, also attach to the different surfaces such as vegetables and food by their adhesions (24). Using alkaline pH and consequently, changes in conformation of bacterial cell surface proteins, bacteria can be eluted from the vegetable surfaces.

In this study, the elution method of viruses from the food surfaces was used to develop a new method for separating food-borne bacteria including *Salmonella enterica* serovar Typhimurium, *Escherichia coli* O157:H7, *Listeria monocytogenes* from the surfaces of lettuce (as a model for ready-to-eat vegetables), so culturing stages were eliminated. Then, the food-borne pathogens were detected by multiplex PCR in a few hours. The proposed method is unique in that it eliminates culturing stages using a new separation method, which permits the rapid detection and identification of target pathogens by multiplex PCR.

## MATERIALS AND METHODS

### Bacterial strains and culture conditions.

Bacterial strains *Salmonella enterica* serovar Typhimurium (PTCC 1709) and *Listeria monocytogenes* (PTCC 1298) were purchased from Persian Type Culture Collection, Iran, and *E. coli* O157:H7 (NCTC 12900) was purchased from National Collection of Type Cultures, UK. The bacteria were grown in Trypticase Soy Broth (Oxoid, UK) at 37°C and then, serial dilutions of strains from 10^0^ to 10^6^ cfu/ml (1 to 1000000 cfu/ml) were prepared using normal saline (0.85 g/l).

### Buffers preparation.

In order to elute bacteria from the surface of lettuce, alkaline distilled water (ADW) and four elution buffers were chosen. The elution buffers were prepared as follows: 1) 0.05 M glycine; 2) 0.05 M glycine-100 mM Tris base-1% (w/v) beef extract (Gly-T-BE); 3) buffer peptone water (BPW); 4) phosphate buffer saline (PBS): 145 mM NaCl; 7.7 mM Na_2_HPO_4_ ; 2.3 mM NaH_2_ PO_4_. The pH of buffers and alkaline distilled water was adjusted to 9, 9.5 and 10 using 5N NaOH (20–23, 25).

### Inoculation of lettuce.

Locally purchased lettuces were cut into pieces of 5 ± 0.2 grams. To sterilize, the pieces overwhelm in 3% sodium hypochlorite solution for 15 min and in order to eliminate extra chloride ions, the samples were moved to sterile distilled water containing some drops of 1% sodium thiosulfate for 2 min. Afterward, the lettuce pieces were washed three times with sterile distilled water and dried under the laminar hood and sterile condition. Then samples were tested for the possible presence of *E. coli* O157:H7, *S. enterica* serovar Typhimurium and *L. monocytogenes* according to ISO standard microbiological methods (26–28).

The strains were inoculated on lettuce as pure and mixed cultures, separately. Lettuce contamination was performed inoculating 100 μl of each serial dilution to the 25 grams sample of lettuce, dried under a laminar flow hood for 60 min under the sterile condition and stored at 4°C overnight (29, 30). For the mixed cultures, 100 μl of each bacterial strain inoculated on lettuce (300 μl in total).

### Effect of alkaline pH on the bacterial survival.

In order to evaluate the effect of alkaline pH on the bacterial survival after elution, the pellets were suspended in 100 μl of sterile normal saline, plated on the general and selective media, incubated at 37°C for 24–48 h and finally the colonies were counted. The used Media were; nutrient agar [Himedia, India (NA), trypticase soy agar [Oxoid, UK (TSA)], sorbitol-MacConky agar [Merck, Germany (SMAK)] for *E. coli* O157:H7; XLD agar [xylose lysine deoxycholate agar (Merck, Germany)] for *S. enterica* serovar Typhimurium; and PALCAM Listeria selective agar [Sigma-Aldrich, Germany (PALCAM)] for *L. monocytogenes*].

### Elution method.

**Fig. 1.**
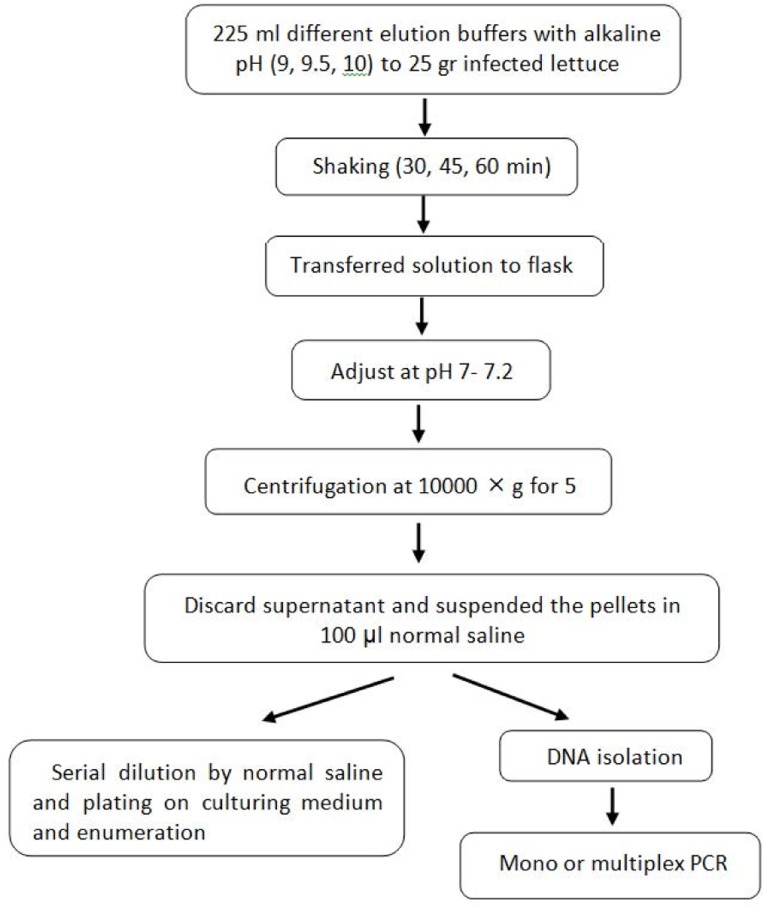
Flow chart of elution method

To optimize the elution method, the inoculated samples were transferred into the stomacher bag containing 225 ml of each buffer solution with different pH (9, 9.5 and 10) and shaked for 30, 45 and 60 min at 150 rpm at room temperature. Then, the elution buffers were transferred into flasks and the pH of buffers was adjusted to pH 7 ± 0.2 with 1N HCl. Next, the samples were centrifuged at 10000 rpm for 5 min and supernatants were discarded. The pellets were suspended in 100 μl of sterile normal saline, plated on the nutrient agar, incubated at 37°C for 24 h and finally, the colonies were counted (Fig. 1). The best pH and time in which the most bacteria were eluted from lettuce surfaces used as a standard elution method for the next steps.

### DNA extraction.

The eluted bacteria were re-suspended in 100 μl of sterile normal saline and used for DNA extraction. DNA extraction was performed on each bacterial strain before and after inoculation to lettuce using Bioneer genomic DNA isolation kit (Bioneer, Korea).

### Mono and Multiplex PCR.

The primer pairs used in this study were shown in Table 1. The target genes were the *rf b* gene (antigen O157 producer) for *E. coli* O157:H7 (31), the *invA* gene (invasion protein A) for *S. enterica* serovar Typhimurium (32), and the *prfA* gene (transcriptional activator of the virulence factor) for *L. monocytogenes* (6). These genes described here are known as the most specific and reliable genetic targets for the above microorganisms. As an internal control, the 16S rRNA gene was targeted in the presence of bacterial DNA (33). An uninoculated control was used in all steps as a negative control and all the experiments were performed three times.

**Table 1.** Primer pairs selected for the single and multiplex PCR

**cteria**	**The size of product**	**Sequence (5′ to 3′)**	**Target Gene**	**Reference**
*E. coli* O157:H7	*rfb*	F: GTG ATG ATG TTG AGT TG R:AGA TTG GTT GGC ATT ACT G	420 bp	31
*Salmonella enterica* serovar Typhimurium	*invA*	F: GTG AAA TTA TCG CCA CGT TCG GGC AA3′ R: TCA TCG CAC CGT CAA AGG AAC C3′	284 bp	32
*Listeria monocytogenes*	*prfA*	F: TCA TCG ACG GCA ACC TCG G R: TGA GCA ACG TAT CCT CCA GAG T	217 bp	6
Universal primer	16s rRNA	27F: AGA GTT TGA TCM TGG CTC AG 1492R: CGG TTA CCT TGT TAC GAC TT	1465 bp	33

### Condition of monoplex PCR.

All monoplex PCR reactions were conducted using GenetBio kit (GenetBio, Korea) in a final volume of 25 μl. Master mix composition was as follows: PCR buffer 10X, 2.5 μl; MgCl _2_ 25 mM, 2.5 μl; *Taq* DNA Polymerase 5 U/μl, 0.2 μl; dNTPs 10 mM, 0.4 μl; F/R primers 10 pmol, 1 μl; extracted DNA as template, 2 μl and distilled water, 15.4 μl.

Thermal cycler (Bio-Rad T100, thermal cycler, Germany) conditions were as follows: predenaturation at 94°C for 5 min; 35 cycles consisting of denaturation at 94°C for 30 s, annealing at 55°C for 30 s, extension at 72°C for 60 s; final elongation at 72°C for 7 min.

### Condition of multiplex PCR.

All multiplex PCR reactions were performed in a final volume of 25 μl using 4 μl of total extracted DNA from mixture of three pathogens as template. Master mix composition was as follows: PCR buffer 10X, 2.5 μl; MgCl_2_ 25 mM, 2.5 μl; *Taq* DNA Polymerase 5U/μl, 0.5 μl; dNTPs 10 mM, 1 μl; EC-F/R primer, 1 μl; SAL-F/R primer, 0.8 μl and LIS-F/R primer, 1 μl (10 pmol concentration of each primer), and distilled water, 8.9 μl.

Thermal cycler conditions were as follows: predenaturation at 94°C for 3min; 35 cycles consisting of denaturation at 94°C for 30 s, annealing at 57°C for 30 s, extension at 72°C for 90 s; final elongation at 72°C for 10 min. PCR products were visualized via gel electrophoresis with 1% agarose gels.

## RESULTS

The aim of this work was the development of an elution method to enhanced rapid detection of foodborne pathogens from ready-to-eat vegetables. For evaluation and development of the elution step, lettuce was used as a model for ready-to-eat vegetables and inoculated with *E. coli* O157:H7, *S. entrica* serovar Typhimurium and *L. monocytogenes*. ADW and four different elution buffers (PBS, 0.05 M Glycine, Gly-T-BE and BPW) were tested for their ability to elute the food-borne bacteria from the surfaces of lettuce.

At first, the elution of bacteria using alkaline pH and their survival after elution were investigated. For recovery, the eluted bacteria were plated on different general and selective culture media. The results showed that alkaline pH has no bactericidal effects and the bacteria can be recovered by culturing on a general media such as nutrient agar (Table 2). Elution at different times, 30, 45 and 60 min were studied; the optimum time to elute the bacteria was 45 min (Table 3). The results shown in Tables 4–6 reveal that BPW and ADW at pH 9.5 for 45 min at 150 rpm and room temperature have high ability to elute the bacteria from the lettuce surface. Both of them were able to recover 99.85–100% of inoculated bacteria at pH 9.5. However, BPW was chosen due to its constant pH during the experiments compare to ADW, which was very variable and the results were not reproducible, therefore, it had not been used in the following experiments.

**Table 2.** The effect of alkaline pH (9.5) and different culturing media on the bacterial survival after elution.

**Bateria Initial inoculation**	**Culture medium**	**Buffers and alkaline water**									

		**PBS**		**Gly**		**Gly-T-BE**		**BPW**		**ADW**	

		**number cfu/ml**	**% recovery**	**number cfu/ml**	**% recovery**	**number cfu/ml**	**% recovery**	**number cfu/ml**	**% recovery**	**number cfu/ml**	**% recovery**
*E. coli*	SMAC	8×10^6^	80	NG	0	NG	0	1.3×10^5^	63.85	NG	0
O157:H7	NA	2.6×10^6^	80.13	3×10^5^	68.5	2.7×10^5^	67.89	1×10^8^	100	1×10^8^	100
1×10^8^ cfu/ml											
*S. entrica* serv.	XLD	2.2×10^6^	79.25	NG	0	NG	0	NG	0	NG	0
Typhimurium	NA	8.9×10^6^	86.75	1×10^6^	75.00	3×10^6^	80.88	1×10^8^	100	9.3×10^7^	99.63
1×10^8^ cfu/ml											
*L. monocytogenes*	PALCAM	1×10^6^	75.00	9.4×10^5^	74.63	5.3×10^5^	71.50	1.8×10^6^	78.19	1.6×10^6^	77.50
1×10^8^ cfu/ml	NA	3×10^6^	81.00	3.5×10^5^	69.25	4.3×10^6^	82.88	1×10^8^	100	1×10^8^	100

NG= not growth; cfu/ml= colony forming units per milliliter; % recovery= log final cfu/ml / log initial cfu/ml × 100; PBS= phosphate buffer saline; Gly= 0.05 M glycin; Gly-T-BE= 0.05 M glysin-100 mM Triss base-1% (w/v) beaf extract; BPW=buffer peptone water; ADW= alkaline distilled water; SMAC= sorbitol-MacConky agar; NA= nutrient agar; XLD= xylose lysine deoxychlate agar; PALCAM= PALCAM Listeria selective agar.

**Table 3.** Percentage of bacterial recovery from lettuce surface using BPW at different incubation time.

**Bacteria**	**Incubation time (min)**	**Inoculated bacterium**		**% recovery**

		**before elution (cfu/ml)**	**After elution (cfu/ml)**	
*E. coli* o157:h7	30		4×10^7^	90.5
	45	2.5×10^8^	2.4×10^8^	99.79
	60		2.4×10^8^	99.79
*S. entrica* serv. Typhimurium	30		5.6×10^7^	88.71
	45	2.5×10^8^	2.45×10^8^	99.89
	60		2.4×10^8^	99.78
*L. monocytogenes*	30		7.6×10^7^	93.83
	45	2.5×10^8^	2.5×10^8^	100
	60		2.5×10^8^	100

cfu/ml= colony forming units per milliliter; % recovery= log final cfu/ml / log initial cfu/ml × 100.

**Table 4.** The numbers of eluted *E. coli* O157:H7 from surface of lettuce by alkaline water and different alkaline buffers.

**Buffers pH**	***E. coli* O157:H7 initial inoculation: 1.5×10^8^ cfu/ml**					

	**pH=9**		**pH=9.5**		**pH=10**	

	**cfu/ml**	**% recovery**	**cfu/ml**	**% recovery**	**cfu/ml**	**% recovery**
PBS	NG	0	2.8×10^6^	78.89	3.3×10^6^	79.7
Gly	1×10^6^	73.34	2.2×10^5^	66.3	NG	0
Gly-T-BE	NG	0	2.35×10^5^	65.68	NG	0
BPW	1.2×10^8^	98.81	1.5×10^8^	100	NG	0
ADW	2.45×10^7^	90.37	1.5×10^8^	100	2×10^6^	77

NG= not growth; cfu/ml= colony forming units per milliliter; % recovery = log final cfu/ml / log initial cfu/ml×100; PBS= phosphate buffer saline; Gly= 0.05 M glycine; Gly-T-BE= 0.05 M glycine-100 mM Tris base-%1 (w/v) beef extract; BPW=buffer peptone water; ADW= alkaline distilled water.

**Table 5.** The numbers of eluted *Salmonella entrica* serovar Typhimurium from surface of lettuce by alkaline water and different alkaline buffers.

**Buffers pH**	***Salmonella entrica* serovar Typhimurium initial inoculation: 1.5×10^8^ cfu/ml**					

	**pH=9**		**pH=9.5**		**pH= 10**	

	**cfu/ml**	**% recovery**	**cfu/ml**	**% recovery**	**cfu/ml**	**% recovery**
PBS	NG	0	1×10^7^	85.62	1.5×10^5^	63.30
Gly	1×10^6^	73.38	1.2×10^6^	74.35	NG	0
Gly-T-BE	NG	0	3.3×10^6^	79.73	NG	0
BPW	6×10^7^	95.15	1.46×10^8^	99.85	1.2×10^8^	98.81
ADW	7×10^7^	96	1.42×10^8^	99.70	5.5×10^6^	82.44

NG= not growth; cfu/ml= colony forming units per milliliter; % recovery= log final cfu/ml / log initial cfu/ml×100; PBS= phosphate buffer saline; Gly= 0.05 M glycine; Gly-T-BE= 0.05 M glycine-100 mM Tris base-%1 (w/v) beef extract; BPW=buffer peptone water; ADW=alkalinedistilled water.

**Table 6.** The numbers of eluted *Listeria monocytogenes* from surface of lettuce by alkaline water and different alkaline buffers.

**Buffers pH**	***Listeria monocytogenes* initial inoculation:1.5×10^8^ cfu/ml**					

	**pH=9**		**pH=9.5**		**pH=10**	

	**cfu/ml**	**% recovery**	**cfu/ml**	**% recovery**	**cfu/ml**	**% recovery**
PBS	NG	0	3.2 ×10^6^	79.70	2.3 ×10^6^	77.80
Gly 0.05 M	1.6 ×10^7^	88.10	5 ×10^4^	57.50	2.3 ×10^6^	77.80
Gly-T-BE	NG	0	1 ×10^6^	73.90	NG	0
BPW	NG	0	1.45 ×10^8^	99.90	NG	0
ADW	3.2 ×10^7^	91.80	1.5 ×10^8^	100	2.6 ×10^7^	90.70

NG= not growth; cfu/ml= colony forming units per milliliter; % recovery= log final cfu/ml / log initial cfu/ml×100; PBS= phosphate buffer saline; Gly= 0.05 M glycine; Gly-T-BE= 0.05 M glycine-100 mM Tris base-%1 (w/v) beef extract; BPW=buffer peptone water; ADW= alkaline distilled water.

After separating and concentration of bacteria, we used mono and multiplex PCR for detection of pathogenic bacteria. To verifying the method and confirm the presence of bacteria after elution, colony counting and single PCR using 16S rRNA gene as internal control was performed (results are not shown). The results demonstrated that multiplex PCR assay is able to identify the presence of three foodborne pathogens with very high sensitivity even at a low-level of a few pathogens <10 CFU per 25 g and 1 CFU/ml for detection of a single pathogen in monoplex PCR.

## DISCUSSION

The growing consumption of fresh and ready-to-eat vegetables has caused an increase in the number of food-borne disease outbreaks that could be hazardous for public health. Traditional culture methods for detection of food-borne pathogens can be laborious and time consuming. Hence, it is necessary to develop a new and rapid method, which could considerably decreases the time consumed.

We investigated the elution of bacteria using alkaline pH and survival of them after elution. The result showed that alkaline pH has no bactericidal effects and the bacteria can be recovered by culturing on a general medium such as nutrient agar. BPW and ADW at pH 9.5 in 45 min have high ability to elute the bacteria from the lettuce surface compared to other buffers. Changing the pH from neutral to alkaline alters the electrical charge from negative to positive on the bacterial surface and consequently separating bacteria from the food surfaces (20). The results also show that altering in electrical charge at pH 9.5 is higher than pH 9 resulting in better elution of bacteria and less bactericidal effect compared with pH 10 ending in higher recovery of bacteria. PWB and ADW were able to recover the most inoculated bacteria. Out of four buffers, however, 0.05 M glycine and Gly-T-BE (0.05 M glycine-100 mM Tris base-1% (w/v) beef extract) buffers had the lowest bacterial recovery showing that buffer composition plays a key role in the bacterial elution and BPW was able to recover of inoculated bacteria. By this new method, we are able to separate and concentrate the pathogenic bacteria from food matrix and eliminate the homogenization of food stuff and the culturing steps.

Immunomagnetic separation (IMS) technology is a promising candidate for food pretreatment systems (10, 12, 15, 18, 34). Antibody-functionalized magnetic beads enable selective separation and concentration of target bacteria from a range of sample matrices. Although commercialized IMS platforms can automatically separate and concentrate immunomagnetic beads with target bacteria, these methods have limitations, such as longer per-enrichment step, small sample volume capacity (18), inability to serotype between 5 to 8% of isolates, incorrect typing due to the loss of bacterial cell surface antigens and being expensive (35). In additional, the capturing ability of the immunomagnetic beads is affected by the presence of inhibitors to specific immunoreactions. Food components such as carbohydrates, proteins and fats normally inhibit the specific binding of the antibody to target molecules (36). Using IMS-mPCR technique, Yang et al. were able to detect *S.* Typhimourium, *L. monocytogenes* and *E. coli* with the detection limit of 10^3^ CFU/g in the artificially contaminated lettuce, tomato and ground beef without any pre-enrichment (12). Ma et al. used IMS-RT-PCR for detection of *Salmonella* spp., *Shigella* spp., and *S. aureus* with the detection limit of 2–9.6 CFU/g, using pre-enrichment for 6 hours (37). These findings showed that the IMS technology required pre-enrichment to have lower limit of detection and good results. Our new elution method can be a promising candidate to replace food per-enrichment and immunomagnetic beads for separating and concentration of pathogenic bacteria.

Many molecular-based methods have been developed for rapid detection of pathogenic bacteria, (12, 13, 30, 31, 36, 37) and, considering the possibility of the coexistence of different pathogens in one sample, multiplex detection and rapid identification of the pathogens in a single analysis is very important and desirable (11).

Polymerase chain reaction (PCR) as a nucleic acid-based method widely used in food-borne pathogens detection. The reported detection limits using mPCR after pre-enrichment have been 1–10 cfu/ml (or per 25 gr) of foodstuffs. Lee et al. (38) and Thapa et al. (2) reported the lowest detection limit of 1 and 10 cfu/ml in food stuff after 12 and 6 hours pre-enrichments, respectively; that is similar to our results, which are without pre-enrichment step. Guan et al. (39) could detect five food-borne pathogens on infected pork with a detection limit of 10^3^ cfu/mL for the simultaneous detection of the five target pathogens and less than 10 cfu/mL for detection of a single pathogen. Such varied results could be due to various PCR inhibitors that can be found in foodstuffs and culture media. Some inhibitors that may affect different steps of the PCR method include: phenolic compounds, fats and glycogen (40). Different methods were used for elimination of the inhibitors such as microfiltration membrane and immunomagnetic separation to improve the detection limits of the PCR assay (10, 14). However, there are also many drawbacks of using this method.

In new elution method, since homogenization of foodstuffs and culturing steps are eliminated, PCR inhibitory factors are in minimal and thus increasing sensitivity. In addition, our new method in combination with the molecular method can be used for rapid detection of the food-borne bacteria and at the same time, for the detection of the food-borne viruses using the same sample.

Other advantages of using our new method are as follows: the minimum equipment requirements, inexpensive, reliable and the most importantly decreasing the time required for the test (preparation time decreases to about 1 hour; overall time decreases to 4 hours). It can be expanded and employed in real samples for the detection of multiple viable pathogens in food products.

## CONCLUSION

The main point of this paper is the elimination of culturing stage and so that decreasing the detection time to a few hours by elution of bacteria from the lettuce surface using alkaline buffers. In addition, a multiplex PCR method for the simultaneous detection of *E. coli* O157:H7, *S. enterica* serovar Typhimurium and *L. monocytogenes* has been described.

The elution of bacteria by alkaline buffers and then performing multiplex PCR was allowed us to set a robust method with high performances to decrease detection time to less than 4 hours compare with other methods, when tested on a complex food system. The sensitivity and robustness of the method proposed together with its ability to perform on a complex food matrix make it a suitable method to be implemented in quality control laboratories for the detection of the target pathogens in food samples.

## References

[1] Elizaquıvel P Aznar R. A multiplex RTi-PCR reaction for simultaneous detection of *Escherichia coli* O157:H7, *Salmonella* spp. and *Staphylococcus aureus* on fresh, minimally processed vegetables. Food Microbiol 2008; 25: 705–713. 18541170 10.1016/j.fm.2008.03.002

[2] Thapa SP Han R Cho JM Hur JH. Multiplex PCR and DNA array for the detection of *Bacillus cereus, Staphylococcus aureus, Listeria monocytogenes, Escherichia coli* O157:H7 and *Salmonella* spp. Targeting virulence-related genes. Ann Microbiol 2013; 63: 725–731.

[3] CDC (Center for disease Control), Investigation Update: Multistate Outbreak of *E. coli* O157:H7 infections associated with In-shell Hazelnuts. 2011 available from: http://www.cdc.gov/ecoli/2011/hazelnuts-4-7-11.html

[4] CDC (Center for disease Control), Cholera and OtherVibrioIllness Surveillance System. 2017 avaiable at: https://www.cdc.gov/nationalsurveillance/pdfs/surveillance-system-overview_covis.pdf

[5] CDC (Center for disease Control), Multistate Outbreak of Human Salmonella Montevideo Infections Linked to Live Poultry. 2012b available at: https://www.cdc.gov/salmonella/montevideo-06-12/signs-symptoms.html

[6] Germini A Masola A Carnevali P Marhelli R. Simultaneous detection of *Escherichia coli* O157:H7, *Salmonella* spp. and *Listeria monocytogenes* by multiplex PCR. Food Control 2009; 20: 733–738.

[7] Yu Q Zhai L Bie X Lu Z Zhang C Tao T Survey of five food-borne pathogens in commercial cold food dishes and their detection by multiplex PCR. Food Control 2016, 59: 862–869.

[8] Ryu J Park SH Yeom YS Shrivastav A Lee SH Kim YR Simultaneous detection of *Listeria* species isolated from meat processed foods using multiplex PCR. Food Control 2013; 32: 659–664.

[9] Jasson V Jacxsens L Luning P Rajkovic A Uyttendaele M. Alternative microbial methods: An overview and selection criteria. Food Microbiol 2010; 27: 710–730. 20630313 10.1016/j.fm.2010.04.008

[10] Zhang QY Zhou WW Zhou Y Wang XF Xu JF. Response surface methodology to design a selective co-enrichment broth of *Escherichia coli, Salmonella* spp. and *Staphylococcus aureus* for simultaneous detection by multiplex PCR. Microbiol Res 2012; 167: 405–412. 22444435 10.1016/j.micres.2012.02.003

[11] Ohk SH Bhunia AK. Multiplex fiber optic biosensor for detection of *Listeria monocytogenes, Escherichia coli* O157:H7 and *Salmonella entrica* from ready-to-eat meat samples. Food Microbiol 2013; 33: 166–171. 23200648 10.1016/j.fm.2012.09.013

[12] Yang Y Xu F Xu H Aguilar ZP Niu R Yuan Y Magnetic nano-beads based separation combined with propidium monoazide treatment and multiplex PCR assay for simultaneous detection of viable *Salmonella Typhimurium, Escherichia coli* O157:H7 and *Listeria monocytogenes* in food products. Food Microbiol 2013; 34: 418–424. 23541211 10.1016/j.fm.2013.01.004

[13] Donhauser SC Niessner R Seidel M. Sensitive quantification of *Escherichia coli* O157:H7, *Salmonella enterica*, and *Campylobacter jejuni* by combining stopped polymerase chain reaction with chemiluminescence flow-through DNA microarray analysis. Anal Chem 2011; 83: 3153–3160. 21417213 10.1021/ac2002214

[14] Vasanth D Pugazhenthi G Uppaluri R. Fabrication and properties of low cost ceramic microfiltration membranes for separation of oil and bacteria from its solution. J Membr Sci 2011; 379: 154–163.

[15] Coklin T Farber JM Parrington LJ Bin Kingombe CI Ross WH Dixon BR. Immunomagnetic separation significantly improves the sensitivity of polymerase chain reaction in detecting *Giardia duodenalis* and *Cryptosporidium* spp. in dairy cattle. J Vet Diagn Invest 2011; 23: 260–267. 21398445 10.1177/104063871102300210

[16] Hagren V Von Lode P Syrjala A Korpimaki T Tuomola M Kauko O An 8-hour system for *Salmonella* detection with immunomagnetic separation and homogeneous time-resolved fluorescence PCR. Int J Food Microbiol 2008; 125: 158–161. 18501459 10.1016/j.ijfoodmicro.2008.03.037

[17] Brandao D Liebana S Pividori MI. Multiplexed detection of food-borne pathogens based on magnetic particles. N Biotechnol 2015; 32: 511–520. 25858812 10.1016/j.nbt.2015.03.011

[18] Lim MC Park JY Park K Ok G Jang HJ Choi SW. An automated system for separation and concentration of food-borne pathogens using immunomagnetic separation. Food Control 2017; 73: 1541–1547.

[19] Croci L Dubois E Cook N Medici D de Schultz AC China B Current methods for extraction and concentration of enteric viruses from fresh fruit and vegetables: Towards international standards. Food Anal Methods 2008; 1: 73–84.

[20] Butot S Putallaz T Sanchez G. Procedure for rapid concentration and detection of enteric viruses from berries and vegetables. Appl Environ Microbiol 2007; 73: 186–192. 17085706 10.1128/AEM.01248-06 PMC1797136

[21] Kim H Kwak I Hwang I Ko G. Optimization of methods for detecting norovirus on various fruit. J Virol Methods 2008; 153: 104–110. 18755218 10.1016/j.jviromet.2008.07.022

[22] Dubois E Hennechart C Deboosere N Merle G Legeay O Burger C Intra-laboratory validation of a concentration method adapted for the enumeration of infectious F-specific RNA coliphage, entrovirus and hepatitis A virus from inoculated leaves of salad vegetables. Int J Food Microbiol 2006; 108: 164–171. 16387377 10.1016/j.ijfoodmicro.2005.11.007

[23] Bahreini M Habibi Najafi MB Bassami MR Yavarmanesh M. Optimization of extraction and concentration methods of enteric viruses from the surface of ready to eat vegetables using MS2 coliphage as a model. J Food Sci Tech 2014; 11: 1–10.

[24] Sapers GM. Efficacy of washing and sanitizing methods for disinfection of fresh fruit and vegetable products. Food Technol Biotechnol 2001; 39: 305–311.

[25] Love DC Casteel MJ Meschke JS Sobsey MD. Methods for recovery of hepatitis A virus (HAV) and other viruses from processed foods and detection of HAV by nested RT-PCR and TaqMan RT-PCR. Int J Food Microbiol 2008; 126: 221–226. 18579246 10.1016/j.ijfoodmicro.2008.05.032

[26] ISO, 11290-2: Microbiology of food and animal feeding stuffs – Horizontal method for the detection and enumeration of *Listeria monocytogenes*– Part 2: Enumeration method. 1998.

[27] ISO 16654: Microbiology of food and animal feeding stuffs – Horizontal method for the detection and enumeration of *Escherichia coli* O157:H7. 2002. 10.1016/j.ijfoodmicro.2018.05.005 29778498

[28] ISO 6579: Microbiology of food and animal feeding stuffs – Horizontal method for the detection of *Salmonella* spp. 2002.

[29] Coudray-Meunier C Fraisse A Martin-Latil S Guillier L Delannoy S Fach P A comparative study of digital RT-PCR and RT-qPCR for quantification of Hepatitis A virus and Norovirus in lettuce and water samples. Int J Food Microbiol 2015; 201: 17–26. 25725459 10.1016/j.ijfoodmicro.2015.02.006

[30] Casteel MG Schmidt CE Sobsey MD. Chlorine disinfection of produce to inactivate hepatitis A virus and coliphage MS2. Int J Food Microbiol 2008; 125: 267–273. 18547665 10.1016/j.ijfoodmicro.2008.04.015

[31] Maurer JJ Schmidt D Petrosko P Sanchez S Bolton L Lee MD. Development of primers to O-antigen biosynthesis genes for specific detection of *Escherichia coli* O157:H7 by PCR. Appl Environ Microbiol 1999; 65: 2954–2960. 10388689 10.1128/aem.65.7.2954-2960.1999 PMC91442

[32] Rahn K De Grandis SA Clarke RC McEwen SA Galán JE Ginocchio C Amplification of an *invA* gene sequence of *Salmonella typhimurium* by polymerase chain reaction as a specific method of detection of *Salmonella*. Mol Cell Probes 1992; 6: 271–279. 1528198 10.1016/0890-8508(92)90002-f

[33] Chiang YC Yang CY Li C Ho YC Lin CK Tsen HY. Identification of *Bacillus* spp., *Escherichia coli, Salmonella* spp., *Staphylococcus* spp. and *Vibrio* spp. with 16S ribosomal DNA-based oligonucleotide array hybridization. Int J Food Microbiol 2006; 107: 131–137. 16386323 10.1016/j.ijfoodmicro.2005.04.028

[34] Wang L Li Y Mustaphai A. Rapid and simultaneous quantitation of *Escherichia coli* 0157:H7, Salmonella, and Shigella in ground beef by multiplex real-time PCR and immunomagnetic separation. J Food Prot 2007; 70: 1366–1372. 17612065 10.4315/0362-028x-70.6.1366

[35] Schrader KN Fernandez-Castro A Cheung WKW Crandall CM Abbott SL. Evaluation of commercial antisera for *Salmonella* serotyping. J Clin Microbiol 2008; 46: 685–688. 18094130 10.1128/JCM.01808-07 PMC2238139

[36] Kim TH Park J Kim CJ Cho YK. Fully integrated lab-on-a-disk for nucleic acid analysis of food-borne pathogenes. Anal Chem 2014; 86: 3841–3848. 24635032 10.1021/ac403971h

[37] Ma K Deng Y Bai Y Xu D Chen E Wu H Rapid and simultaneous detection of *Salmonella, Shigella*, and *Staphylococcus aureus* in fresh pork using a multiplex real-time PCR assay based on immunomagnetic seperation. Food Control 2014; 42: 87–93.

[38] Lee N Kwon KY Oh SK Chang HJ Chun SH Choi SW. A multiplex PCR assay for simultaneous detection of *Escherichia coli* O157:H7, *Bacillus cereus, Vibrio parahaemolyticus, Salmonella* spp., *Listeria monocytogenes*, and *Staphylococcus aureus* in Korean ready-to-eat food. Foodborne Pathog Dis 2014; 11: 574–580. 24796416 10.1089/fpd.2013.1638

[39] Guan ZP Jiang Y Gao F Zhang L Zhou GH Guan ZJ. Rapid and simultaneous analysis of five food-borne pathogenic bacteria using multiplex PCR. Eur Food Res Technol 2013; 237: 627–637.

[40] Garrido A Chapela MJ Román B Fajardo P Vieites JM Cabado G. In-house validation of a multiplex real-time PCR method for simultaneous detection of Salmonella spp., *Escherichia coli* O157:H7 and *Listeria monocytogenes*. Int J Food Microbiol 2013; 164: 92–98. 23624537 10.1016/j.ijfoodmicro.2013.03.024

